# Successful bone marrow transplantation in a patient with Diamond-Blackfan anemia with co-existing Duchenne muscular dystrophy: a case report

**DOI:** 10.1186/1752-1947-5-216

**Published:** 2011-06-04

**Authors:** Velu Nair, Satyaranjan Das, Ajay Sharma, Sanjeevan Sharma, Jasmeet Kaur, DK Mishra

**Affiliations:** 1 Department of Haematology & Bone Marrow Transplantation, Army Hospital (Research & Referral), Delhi Cantt-110010, India; 2 Department of Microbiology, Army Hospital (Research & Referral), Delhi Cantt-110010, India; 3 Department of Pathology, Army Hospital (Research & Referral), Delhi Cantt-110010, India

## Abstract

**Introduction:**

Diamond-Blackfan anemia and Duchenne muscular dystrophy are two rare congenital anomalies. Both anomalies occurring in the same child is extremely rare. Allogeneic hematopoietic stem cell transplantation is a well-established therapy for Diamond-Blackfan anemia. However, in patients with Duchenne muscular dystrophy, stem cell therapy still remains experimental.

**Case presentation:**

We report the case of a nine-year-old boy of north Indian descent with Diamond-Blackfan anemia and Duchenne muscular dystrophy who underwent successful allogeneic hematopoietic stem cell transplantation. He is transfusion-independent, and his Duchenne muscular dystrophy has shown no clinical deterioration over the past 45 months. His creatine phosphokinase levels have significantly decreased to 300 U/L from 14,000 U/L pre-transplant. The patient is 100% donor chimera in the hematopoietic system, and his muscle tissue has shown 8% to 10.4% cells of donor origin.

**Conclusion:**

Our patient's Diamond-Blackfan anemia was cured by allogeneic hematopoietic stem cell transplantation. The interesting clinical observation of a possible benefit in Duchenne muscular dystrophy cannot be ruled out. However, further clinical follow-up with serial muscle biopsies and molecular studies are needed to establish this finding.

## Introduction

Diamond-Blackfan anemia (DBA) is a rare congenital hypoplastic anemia that usually presents in infancy and childhood. The incidence of DBA is five to 10 per one million births, and more than 600 cases have been reported worldwide. The exact cause is unclear, but the problem is a defect in one of the early steps in erythropoiesis (red blood cell (RBC)) production. In about 15% of affected children, there is a defect within a gene called *RPS19 *(ribosomal protein S19) [1]. In most cases, occurrence is sporadic, but in subsequent generations, inheritance is usually autosomal dominant. Nearly 90% of these children are transfusion-dependent by 18 months of age. About 60% of patients initially respond to drugs such as steroids, androgens and cyclosporine, though one-half of these initial responders become refractory to these medications later [2]. The only curative treatment for DBA is allogeneic hematopoietic stem cell transplant (allo-HSCT), which has an 85% success rate [3-5] and is offered to patients who do not respond to or become refractory to drug therapy.

Duchenne muscular dystrophy (DMD), which is caused by mutation of the dystrophin gene, is the most common and severe form of muscular dystrophy. This disorder is marked by progressive loss of muscle function which begins in the lower limbs and later occurs in the arms, neck and other areas. DMD occurs in approximately one of 3500 male live births and is inherited in an X-linked recessive pattern [6]. Terminally, the respiratory muscles are also involved. The calf muscles initially grow larger (pseudo-hypertrophy) but are eventually replaced by fat and connective tissue. The muscle fibers shorten because of fibrosis. The diagnosis is suspected when the creatine phosphokinase (CPK) levels are highly elevated and is confirmed by muscle biopsy. There is no known cure for DMD, and treatment is only supportive. However, recently there have been anecdotal reports of benefit following allo-HSCT treatment [7-10].

## Case presentation

DBA and DMD are two rare congenital anomalies. Both anomalies occurring in the same child is extremely rare. We report such an unusual case of a nine-year-old boy of north Indian descent, the third of four siblings born to healthy, non-consanguineous parents with a family history of DMD in a maternal first cousin. The boy was presented to our hospital at the age of eight months with progressive pallor and failure to thrive. He was diagnosed with DBA on the basis of peripheral blood showing normocytic, macrocytic anemia and reticulocytopenia with normocellular bone marrow showing erythroblastopenia. Though initially steroid-responsive, the boy later became unresponsive to both steroids and cyclosporine. By the fourth year of life, he was completely transfusion-dependent, and iron chelation was started only in the sixth year of life with intravenous desferrioxamine when his serum ferritin level was found to be 5100 ng/mL.

The patient had normal milestones until the fourth year of life. Thereafter he developed lower-limb weakness and calf muscle pseudohypertrophy. The weakness was progressive in nature, and by the age of five years the boy required support to climb stairs. His CPK level was high (5939 U/L), and his electromyogram was consistent with myopathy. He was diagnosed with DMD. A muscle biopsy was not done as the parents were unwilling to allow their son to undergo the procedure at that time. At this stage, the child was referred to our center for allo-HSCT treatment in view of his transfusion-dependent DBA with hyperferritinemia. The diagnosis was reconfirmed at our center. He also had dysmorphic facial features. However, *RPS19 *genetic studies could not be done.

The child had an unaffected elder brother who was a human leukocyte antigen (HLA)-identical match (6/6 antigen match) and underwent myeloablative allo-HSCT treatment. The conditioning regimen comprised busulphan (16 mg/kg over four days), cyclophosphamide (200 mg/kg over four days) and equine anti-thymocyte globulin (Atgam; Pfizer(Pharmacia & Upjohn company, a subsidiary of Pharmacia Corporation

Kalamazoo, Michigan 49001, USA) (90 mg/kg over three days). There was a minor blood group mismatch, with the recipient being in the B+ve group and the donor being in the O+ve blood group. Granulocyte colony-stimulating factor primed bone marrow was harvested from the donor while the donor was under general anesthesia. Plasma depletion was done using a Cryofuge 6000i centrifuge(by Heraeus Instruments made in Germany and supplied by Kendro Labarotories (India)) at 3000 rpm, and plasma-depleted marrow was infused with a cell dose of 6 × 10^8 ^mononuclear cells (MNCs)/kg body weight. Graft-versus-host disease (GVHD) prophylaxis consisted of standard dose methotrexate and cyclosporine. Neutrophil and platelet engraftment occurred on day+11 and day+16, respectively. RBC engraftment was confirmed with a change of blood group on day+90. The child has been transfusion-free since 2 months post-transplant, and his serum ferritin levels have been reduced to 600 ng/mL with regular phlebotomies. The boy's blood counts were normal 45 months after allo-HSCT treatment: hemoglobin 13.5 g/dL, reticulocyte count 1%, white blood cell count 6.0 × 10^3^/μL with 70% neutrophils and platelet count 1900 × 10^3^/μL.

This patient was evaluated by a neurologist pre-transplant and periodically post-transplant. The patient was wheelchair-bound pre-transplant, and, 45 months post-transplant, there has been no clinical deterioration whatsoever in the boy's motor power. He continues to be wheelchair-bound and is able to sit on his own for more than three hours at a stretch. The CPK level pre-transplant had been in the range of 9000 to 14,000 U/L and showed a declining trend within four weeks of transplantation, reaching a nadir of 300 U/L by six months post-transplantation. The muscle biopsy, which was done twice at 730 and 1250 days post-HSCT, respectively, revealed mixed donor chimerism with 8% to 10% cells of donor origin. Sequential chimerism studies using whole blood established trilineage engraftment with 100% donor chimerism. There were no post-transplant complications in the form of sepsis, hepatic veno-occlusive disease or acute or chronic GVHD.

Chimerism analysis performed on recipient peripheral blood samples on day+30, day+90, day+365, day+730 and day+1250 revealed complete donor chimerism post-transplant. A muscle biopsy was performed twice post-bone marrow transplantation (BMT) at days 730 and 1250 to study histopathology, dystrophin expression and chimerism status. Immunostaining for dystrophin I was reduced, dystrophin II and III were absent and there was up-regulation of utrophin.

**Figure 1:**
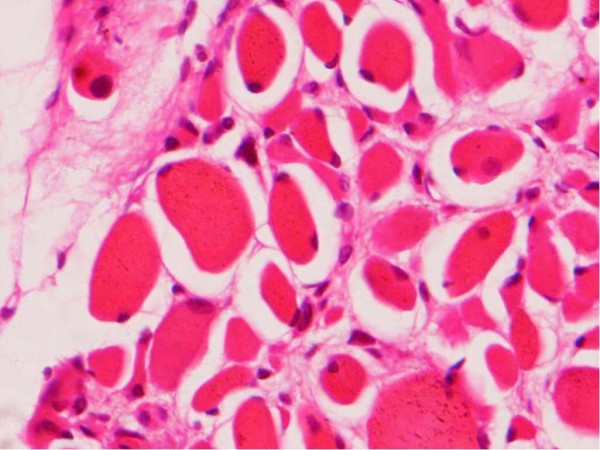
**Hematoxylin and eosin-stained section showing post-transplantation muscle biopsy**. Image shows a cross-sectional view of muscle fibers of varying sizes that have predominantly peripheral nuclei with a few fibers displaying central nuclei and regenerative changes. There is scanty intervening stroma.

Chimerism was performed to monitor donor cell engraftment in the recipient peripheral blood and was also used to detect donor cells in the muscle biopsy from the recipient. Chimerism was carried out using recipient's hair follicle as the pre-transplant sample [11], peripheral whole blood as the post-HSCT sample, and whole blood sample from the donor. Blood was collected in ethylenediaminetetraacetic acid vacutainers. DNA extraction from whole blood was carried out using the QIAamp DNA Blood Mini Kit (Qiagen, Hilden, Germany) according to the manufacturer's instructions. Preparation of hair follicles was done with 10 full-length hairs with roots plucked from different areas of the recipient's scalp. The presence of the hair bulb was visually confirmed. Careful washing was done to minimize the risk of blood contamination by rinsing in normal saline. The QIAamp DNA Mini Kit was used for DNA extraction from hair roots and muscle biopsy. The muscle biopsy specimen was preserved in 10.4% formaldehyde for routine histopathological processing and stained with hematoxylin and eosin (Figure 1).

**Figure 2:**
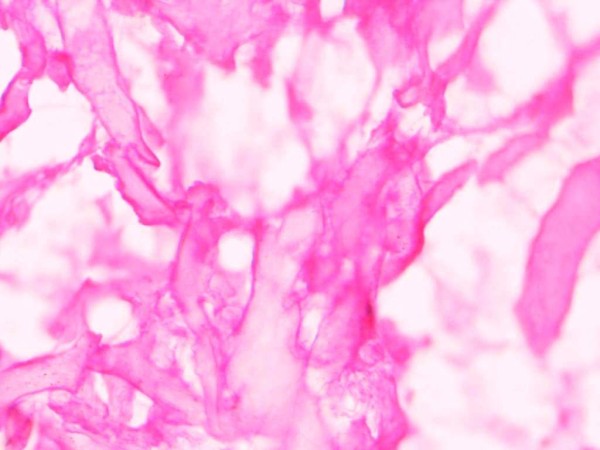
**Hematoxylin and eosin-stained cryosections of serially cut biopsies of muscle tissue that was homogenized and washed with saline prior to extraction of tissue DNA**. No evidence of mononuclear cell contamination of the muscle fibers was noted.

The fragment of muscle biopsy taken for chimerism analysis was washed in saline seven times to remove all traces of RBCs and peripheral blood MNCs (PBMCs). The muscle tissue was homogenized and further rinsed three times in saline, following which a frozen section of the homogenized muscle was taken to check for any contamination with PBMCs (Figure 2). Genomic DNA was extracted from the muscle tissue cryosections serially cut from biopsies using the QIAamp DNA Blood Mini Kit according to the manufacturer's instructions.

**Figure 3:**
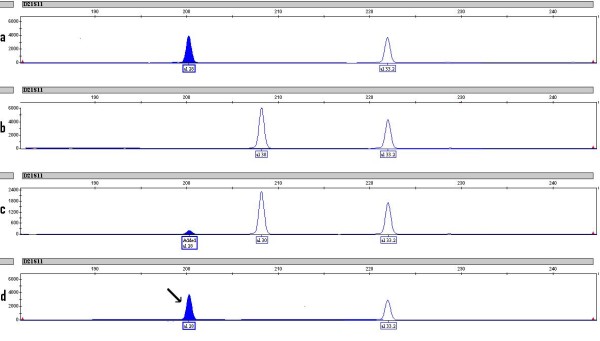
**Microsatellite analysis of locus D21S11 in (a) donor peripheral blood sample showing peaks at allele 28 and 33.2, (b) recipient hair follicle representing patient pre-transplant DNA with allele 30 and 33.2, (c) recipient muscle biopsy sample on D+730 showing mixed chimerism with alleles 28, 30 and 33.2 and (d) recipient peripheral blood sample on D+730 showing complete donor chimerism**.

Chimerism of all samples was performed using 16 short tandem repeat markers (D8S1179, D21S11, D7S820, CSF1PO, D3S1358, THO1, D13S317, D16S539, D2S1338, D19S433, vWA, TPOX, D18S51, AMEL, D5S818 and FGA) labeled with four types of fluorescent dyes (6FAM, VIC, NED and PET). A multiplex polymerase chain reaction assay was performed in a final reaction volume of 25 μL containing 10.5 μL of reaction mixture, 5.5 μL of primer mix, 0.5 μL of AmpFl STR Identifier kit (Applied Biosystems, Foster City, CA, USA) and 10 μL of DNA at a concentration of 0.2 ng/μL. Cycling parameters were optimized as follows: 95°C for 11 minutes (one hold), 94°C for 60 seconds, 59°C for 60 seconds, 72°C for 60 seconds and 28 cycles, 60°C for 60 minutes (two holds) in a GeneAmp PCR System 9700 (Applied Biosystems). Denaturation was performed for five minutes at 95°C using Hi-Di Formamide and GeneScan 500 LIZ Size Standard (both from Applied Biosystems). The amplicon was resolved by performing capillary electrophoresis using the ABI Prism 3100-Avant Genetic Analyzer System (Applied Biosystems) and analyzed using the Applied Biosystems *Gene mapper TM software v 3.5, Foster City CA 94404, USA*. To determine the fraction of donor cells in the recipient's peripheral blood and muscle biopsy samples post-transplantation, the informative markers were identified and the percentage of donor cells was estimated. While the post-transplant recipient peripheral blood sample displayed complete donor chimerism, the muscle biopsy sample showed mixed donor chimerism with 8% to 10.4% donor cells on days 730 and 1250, respectively (Figure 3), suggesting the presence of donor-derived cells in the recipient's muscle.

## Discussion

Allo-HSCT is the only available curative treatment for DBA. The first "successful" allo-HSCT treatment of DBA was reported in 1976 [12]. The patient died, but hematopoietic engraftment from donor bone marrow confirmed DBA as a transplantable disease. Since the initial case, more than 70 transplants, the majority of which involved from HLA-matched sibling donors, have been reported in the literature [13,14]. The outcomes of patients who undergo alternative donor stem cell transplantation are significantly inferior to those of HLA-matched sibling donors [14].

DMD has no known cure. Experiments with stem cells on animals with DMD have been encouraging. In *mdx *mouse models of DMD, allo-HSCT from normal mice led to incorporation of donor-derived nuclei into muscle and partial restoration of dystrophin expression in the affected muscle. The canine model of DMD (canine X-linked muscular dystrophy (CXMD)) has shown a clinical course very similar to that of human DMD. In a study of seven CXMD dogs that underwent allo-HSCT from non-affected littermates, there was no increase in the number of dystrophin-positive fibers or in the amount of wild-type dystrophin RNA post-transplantation compared with pre-transplantation levels. However, another canine study demonstrated that allo-HSCT provides an immune-tolerant platform for myoblast transplantation from freshly isolated muscle-derived cells from the same HSCT donor [15].

The first successful allo-HSCT in a patient with DMD was reported in 2002. That patient with DMD was diagnosed at 12 years of age and underwent BMT at one year of age for X-linked severe combined immunodeficiency syndrome. Analysis of muscle biopsies revealed the presence of donor nuclei within a small number of muscle myofibers (0.5% to 0.9%). The discovery of the donor's mesenchymal cells in the patient's muscle tissue and bone marrow 13 years after the transplant raised the hope that BMT may play a role in the treatment of DMD [7]. Two case reports from China of unrelated allogeneic umbilical cord HSCT in young boys with advanced DMD have shown some improvement post-transplantation. This improvement has been reported both clinically by improved motor activity and in laboratory parameters seen by reduction in CPK levels, increase in dystrophin-positive muscle fibers and reduction of the defective gene transcripts measured by PCR [9,10].

## Conclusion

To the best of our knowledge, we report the first case of a boy with two rare genetic disorders, DBA and DMD, who successfully underwent myeloablative allo-HSCT, which cured his DBA and might have had a positive effect on his DMD. Clinically, the course of his DMD has seemed milder and nearly static over the past 45 months as compared to his first cousin, who also has DMD and by the same age was unable to sit without support. In addition, CPK levels showed drastic reduction, and chimerism studies revealed 8% and 10.4% donor cells in the patient's skeletal muscle at days 730 and 1250, respectively.

Though this percentage of donor cells was small, the presence of these cells in the patient's muscle tissue indicates the possibility of transdifferentiation of hematopoietic donor stem cells to skeletal muscle myocytes in the patient. However, no dystrophin expression was noted in the muscle tissue, which could possibly be due to the low level of transdifferentiation. Though this transplant was done primarily for DBA, it raises the interesting possibility of allo-HSCT's being beneficial in the treatment of DMD, which is an otherwise incurable disease with 100% mortality. However, further clinical follow-up with serial muscle biopsies and molecular studies is needed to document the extent and duration of mixed chimerism in skeletal muscle in this patient. The purpose of this case report is to describe this interesting observation of a possible benefit in DMD and not to suggest HSCT as a modality of treatment until further studies show an unequivocal benefit, given the inherent risks associated with HSCT.

## Consent

Written informed consent was obtained from the patient's parent for publication of this case report and accompanying images. A copy of the written consent is available for review by the Editor-in-Chief of this journal.

## Competing interests

The authors declare that they have no competing interests.

## Authors' contributions

BVN is the first author and is responsible for the conception and design of the case report. Contributing authors SD, AS, SS, JK and DKM made substantial contributions to the design of the manuscript and the acquisition, analysis and interpretation of the data.

## Authors' information

VN is a consultant in Medicine & Clinical Haematology and was Head of the Department of Medicine and Head of the Department of Haematology & BMT, Army Research & Referral Hospital, New Delhi, India, which is the premier institution of the Indian Armed Forces. He was also the Dean of the Army College of Medical Sciences, New Delhi, India. Presently, he is Head of the Department of Medicine at the Armed Forces Medical College in Pune, India. He is a well-known clinician, researcher and BMT physician with over 25 years of experience. He has written more than 150 publications in various national and international journals, including *Bone Marrow Transplantation*, *Acta Haematologica*, *Blood*, *Journal of the Association of Physicians of India*, *Indian Pediatrics*. Medical Journal of Armed Forces of India and Indian Journal of Haematology & Blood Transfusion among others. VN has done two International Cancer Technology Transfer Fellowships under the technology transfer program of the International Union Against Cancer, Geneva, at King's College & Hospital, London in 2004, and at Stanford University School of Medicine, Stanford, CA, USA, in 2008. He is also the former President of the Indian Society of Haematology & Transfusion Medicine and a fellow of the American College of Physicians and the Indian Academy of Medical Sciences.

All other contributing authors are either professors or associate professors in the specialties mentioned on the title page and have significant experience and have written numerous publications.
